# General anesthesia and depth of anesthesia (DoA) evaluation methods in laboratory animals: a comprehensive review

**DOI:** 10.1007/s11259-026-11126-2

**Published:** 2026-03-17

**Authors:** Tülin Altınoluk, Hasan Kazdağlı

**Affiliations:** Vocational School of Health Services, Izmir University of Economics, Izmir, Türkiye

**Keywords:** Laboratory animals, Anesthetics, Depth of anesthesia, EEG monitoring, Heart rate variability

## Abstract

In preclinical research, general anesthesia is essential for humane and feasible procedures but profoundly modulates autonomic, cardiovascular, neurological, and biochemical systems, risking bias in experimental outcomes. In this review our aim was to synthesize current knowledge on commonly used general anesthetics in laboratory animals, their mechanisms and protocols across species (mice, rats, rabbits, pigs), and evidence-based methods to evaluate depth of anesthesia (DoA). Injectable agents (ketamine with α_2_-agonists, barbiturates, propofol) and inhalational agents (isoflurane, sevoflurane, desflurane) act primarily via NMDA antagonism or GABA-A/glycine modulation, with distinct profiles for analgesia, hemodynamics, respiration, and recovery. Species-specific dosing and routes are summarized for small rodents, rabbits, and pigs, including practical considerations (e.g., thermoregulation, airway management, malignant hyperthermia risk). DoA assessment spans traditional reflex-based scoring and advanced monitoring. EEG, raw and processed indices (e.g., BIS), offers continuous cortical information but requires species-specific validation and cautious interpretation. Autonomic indicators (heart rate, blood pressure, respiratory patterns) and heart-rate variability provide complementary, noninvasive signals yet are confounded by drugs, ventilation, and surgical stimuli. Anesthetic choice is a major experimental variable. Reliable practice demands multimodal DoA monitoring that integrates reflexes, physiologic trends, and, where feasible, EEG, alongside rigorous, species-adapted protocols and transparent reporting. Priorities include validated EEG algorithms for nonhuman species, standardized autonomic indices, and broader adoption of awake models when compatible with scientific aims. These strategies will improve animal welfare and enhance the reproducibility and interpretability of preclinical findings.

## Introduction

General anesthesia is an indispensable component of experimental procedures in laboratory animals, ensuring adequate sedation, analgesia, and muscle relaxation while facilitating humane handling and invasive interventions (Kazdağli et al. 2022). Beyond its intended effects on consciousness and nociception, general anesthesia exerts profound influences on multiple physiological systems, among which the autonomic nervous system (ANS) holds a pivotal role in maintaining homeostasis (Wehrwein et al. 2016). The ANS regulates cardiovascular, respiratory, thermoregulatory, and gastrointestinal functions through intricate sympathetic and parasympathetic pathways, and its modulation by anesthetic agents can lead to transient or persistent alterations in these processes (Göthert 1982). The altered ANS function also causes indirect cardiovascular effects additional to direct effects on the cardiovascular system (Kazdağli et al. 2022).

The cardiovascular and autonomic functions are affected significantly by anesthetics, and responses to certain interventions are quite different between unanesthetized and anesthetized animals (Shimokawa et al. 1998; Kannan et al. 1989; Hegarty et al. 1995). For example, the increase in renal sympathetic nerve activity (RSNA) by an electrical stimulation of the hypothalamic paraventricular nucleus (PVN) in unanesthetized rats is attenuated remarkably under pentobarbital anesthesia (Kannan et al. 1989). The pressor response caused by a microinjection of noradrenaline to the nucleus tractus solitarius in unanesthetized rats is attenuated by pentobarbital anesthesia and is abolished by urethane anesthesia (Vlahakos et al. 1985). Therefore, it is important to elucidate how anesthesia per se affects the activity of the autonomic nervous system and cardiovascular system.

The use of anesthetics in laboratory animals causes various physiological and biochemical alterations that can profoundly impact experimental data interpretation (Hernández-Godínez et al. 2019). Understanding the implications of anesthesia selection, including depth and type of agent, is crucial for achieving reliable and reproducible research outcomes (Kazdağli et al. 2022). For instance, Kirihara et al. demonstrated notable variances in oxygen saturation among different strains of rats when administered an anesthetic mixture of medetomidine, midazolam, and butorphanol (Kirihara et al. 2016). Their findings suggest that the choice of anesthetic can induce differing anesthetic depths, which may bias experimental results based on the physiological condition of the animal during study (Kirihara et al. 2016). This is further corroborated by Tsukamoto et al., who noted that proper anesthesia selection is imperative, as inadequate depth can compromise animal welfare and affect the reliability of physiological measurements in research settings (Tsukamoto et al. 2015a, b).

Moreover, the neurological effects of anesthetics complicate their use in experimental design, particularly in studies related to brain function. Brown (2010) emphasized that anesthetics produce a condition that resembles a ' temporary, reversible coma’ a state that diverges significantly from natural physiological processes (Brown 2010). The shift in brain-state dynamics necessitates a reevaluation of experimental paradigms, as traditional models relying on anesthetized animals may yield data that misrepresents normal brain activity (Zhang 2022). Recent movements in the neuroscience community advocate for the use of awake animal models, highlighting a fundamental shift toward methodologies that better reflect the physiological realities of un-anesthetized states (Zhang 2022).

The selection of anesthetic agents also has implications for the biochemical assays utilized in laboratory research. Gibbs et al. (2019) elucidated the effects of common anesthetics on urinary biomarkers indicative of kidney injury in mice, emphasizing the importance of understanding how these agents can alter biomarker levels. This knowledge is essential to avoid confounding factors that could mislead interpretations of experimental results (Gibbs et al. 2019). Furthermore, the monitoring of physiological changes under anesthesia, as outlined by Tremoleda et al., underscores the necessity for careful consideration regarding how anesthetic agents influence experimental outcomes and animal welfare in imaging studies (Tremoleda et al. 2012).

Selecting an appropriate anesthetic regimen should therefore be predicated on a thorough understanding of both the anticipated physiological effects on the animal and the specific requirements of the experimental protocol. While anesthesia can influence outcomes beyond immediate physiological changes, most modern anesthetic agents exhibit low systemic toxicity, and potential long-term effects are primarily relevant to specific research domains, such as neuropharmacology and brain biochemistry, rather than across all biomedical applications (Ochiai et al. 2018; Navarro et al. 2021). In routine preclinical practice, the primary concern is to minimize anesthesia-related complications through careful monitoring and timely corrective interventions.

It is also important to acknowledge that the availability and implementation of anesthetic agents and monitoring techniques vary considerably across countries and research settings. In some regions, legal restrictions, limited access to modern anesthetics and analgesics (e.g. opioids, ketamine, benzodiazepines), and insufficient infrastructure may prevent adherence to advanced anesthesia and monitoring standards in laboratory animals. Under such constraints, the expertise and training of veterinarians in anesthesia become particularly critical, as does the judicious use of balanced protocols incorporating regional and local anesthesia techniques to optimize analgesia and minimize physiological stress. Recognizing these real-world limitations is essential to ensure that recommendations on laboratory animal anesthesia remain globally relevant and practically applicable.

In light of these considerations, this review aims to provide a comprehensive synthesis of current knowledge regarding the effects of general anesthesia in laboratory animals, with particular emphasis on its physiological, neurological, and biochemical implications. By systematically evaluating commonly used anesthetic agents and their impact on experimental outcomes, our objective is to guide researchers in making informed decisions about anesthetic selection, optimize animal welfare, and improve the reliability and reproducibility of preclinical research. This review also highlights methodological challenges and future directions, thereby serving as a practical resource for investigators across diverse fields of biomedical science.

## Commonly used general anesthetics and mechanisms of action

General anesthetics employed in laboratory animals can be broadly divided into injectable and inhalational agents. Each group has characteristic pharmacokinetic and pharmacodynamic properties, molecular targets, and systemic effects that may influence both animal welfare and experimental outcomes. A clear understanding of their mechanisms of action is critical for appropriate anesthetic selection and for interpreting research findings (Oh and Narver 2024).

### Injectable anesthetics

#### Barbiturates

Barbiturates such as thiopental and pentobarbital have been widely used for decades in laboratory research. Their primary mechanism involves potentiation of γ-aminobutyric acid type A (GABA-A) receptor-mediated chloride influx, resulting in hyperpolarization and depression of neuronal excitability (Eger et al. 1997). At higher doses, they can directly activate GABA-A receptors independent of GABA. These agents provide rapid induction of hypnosis and anesthesia but have limited analgesic effects. They also cause dose-dependent cardiovascular and respiratory depression, which may confound experimental readouts, particularly in cardiovascular studies (Eger et al. 1997).

#### Ketamine

Ketamine is one of the most frequently used injectable anesthetics in small animal research. It acts as a non-competitive antagonist of the N-methyl-D-aspartate (NMDA) receptor, thereby blocking excitatory glutamatergic neurotransmission (Hirota and Lambert 1996). This produces a “dissociative anesthesia,” characterized by profound analgesia, amnesia, and catalepsy. Unlike barbiturates, ketamine generally preserves respiratory function and airway reflexes, but it increases sympathetic outflow, leading to elevated heart rate and blood pressure (Hirota and Lambert 1996). In rodents, ketamine is often combined with α_2_-adrenergic agonists such as xylazine or medetomidine to improve muscle relaxation and deepen anesthesia

#### ***α***_***2***_***-Adrenergic agonists***

Agents such as xylazine, medetomidine, and dexmedetomidine act primarily via presynaptic α_2_-adrenergic receptors to inhibit norepinephrine release, thereby reducing sympathetic tone (Virtanen et al. 1988). Their pharmacological profile includes sedation, analgesia, and muscle relaxation, and they are most commonly used as adjuncts to dissociative or injectable anesthetic protocols rather than as sole anesthetic agents (Virtanen et al. 1988).

Importantly, α_2_-agonists differ in their α_1_:α_2_ receptor selectivity, which has direct implications for both efficacy and adverse effect profiles. Xylazine exhibits relatively low α_2_ selectivity (α_1_:α_2_ ratio approximately 1:160), whereas medetomidine (~ 1:1620) and dexmedetomidine (~ 1:1620–2000) are substantially more α_2_-selective. Higher α_2_ selectivity is associated with more predictable sedation and analgesia at lower doses, as well as reduced α_1_-mediated excitatory and cardiovascular effects. Nevertheless, even highly selective agents can induce dose-dependent bradycardia, hypotension, and respiratory depression, necessitating vigilant physiological monitoring, particularly in young or compromised animals (Virtanen et al. 1988).

#### Propofol

Propofol is a short-acting intravenous anesthetic with rapid onset and recovery. Its mechanism involves enhancement of GABA-A receptor-mediated inhibitory neurotransmission, resulting in sedation and hypnosis (Trapani et al. 2000 a). The short half-life of propofol makes it attractive for imaging studies and procedures requiring quick adjustments in anesthetic depth. However, it induces dose-dependent hypotension and respiratory depression, limiting its use in prolonged rodent anesthesia without ventilatory support (Trapani et al. 2000a).

### Inhalational anesthetics

Inhalational anesthetics are critical tools in veterinary medicine for laboratory animals (Oh and Narver 2024). These agents are preferred due to their ability to allow precise control over anesthetic depth and rapid recovery once administration ceases (Hohlbaum et al. 2017). Isoflurane is widely recognized as the most commonly employed inhalational anesthetic agent in laboratory settings, largely due to its favorable pharmacokinetics and safety profile in small mammals such as rats and mice (Tsukamoto et al. 2015a, b). Sevoflurane and desflurane are increasingly used as alternative inhalational anesthetics in laboratory animal studies and exhibit more favorable pharmacokinetic properties than isoflurane, particularly with respect to faster induction and recovery. Nevertheless, isoflurane remains the most commonly used volatile anesthetic in preclinical research, largely due to its lower cost, widespread availability, and long-standing familiarity in laboratory animal protocols, rather than any superiority in onset or offset characteristics (Brunson 2008).

In addition to their pharmacological and physiological effects, inhalational anesthetics pose important occupational health considerations due to exposure to waste anesthetic gases (WAGs). Chronic exposure to volatile anesthetics such as isoflurane, sevoflurane, and desflurane has been associated with adverse health effects in personnel, including neurological symptoms, reproductive disturbances, and potential genotoxicity, particularly in inadequately ventilated laboratory environments (Deng et al. 2018; Sessler and Badgwell 1998). Consequently, effective control of WAGs is a critical component of safe inhalational anesthesia practice in laboratory animal facilities.

Appropriate engineering controls, including active or passive scavenging systems connected to anesthesia circuits, adequate room ventilation, and the use of well-fitted masks or sealed induction chambers, are essential to minimize environmental contamination. Regular equipment maintenance, leak testing, and proper training of personnel further reduce exposure risk (NIOSH 2007). Compliance with institutional guidelines and occupational safety standards is particularly important in small-animal laboratories, where high fresh gas flows and frequent mask anesthesia may substantially increase ambient anesthetic concentrations. Incorporating WAG management into routine anesthesia protocols enhances personnel safety without compromising anesthetic efficacy or animal welfare.

#### Isoflurane

Isoflurane is a volatile anesthetic widely used in clinical settings for its ability to induce and maintain anesthesia. Its mechanism of action primarily involves the modulation of GABA-A receptors, which play a crucial role in mediating inhibitory neurotransmission in the brain (Hall et al. 2004). Research indicates that isoflurane enhances the efficacy of GABA-A receptor currents, leading to increased inhibitory postsynaptic potentials (IPSPs) across various neural circuits (Hall et al. 2004).

One of the key findings regarding isoflurane’s action is its enhancement of GABA-A receptor activity (Hall et al. 2004). Isoflurane has been shown to increase the currents generated by GABA-A receptors in a concentration-dependent manner. This potentiation can depend on the specific subunit composition of the GABA-A receptors, as certain mutations in receptor subunits can diminish the anesthetic effects of isoflurane (Hall et al. 2004; Topf et al. 2003). Studies have demonstrated that isoflurane prolongs the decay of IPSPs, likely through modulation of desensitized GABA-A receptor states, permitting enhanced signaling during synaptic activity under anesthetic conditions (Hall et al. 2004; Topf et al. 2003).

#### Sevoflurance

Sevoflurane is a widely utilized inhalational anesthetic known for its rapid onset and recovery characteristics in clinical practice (Benković et al. 2023). Its mechanisms of action, although not completely understood, involve multiple pharmacological pathways impacting central nervous system (CNS) function primarily through modulation of ion channels and neurotransmitter systems (Benković et al. 2023).

One of the fundamental mechanisms of sevoflurane’s anesthetic action is its interaction with various ion channels. Research indicates that sevoflurane acts on glycine receptors and GABA-A receptors, enhancing inhibitory neurotransmission and reducing excitatory neurotransmission. This dual action leads to a general depression of CNS activity, which is essential for achieving anesthesia (Benković et al. 2023). Specifically, sevoflurane’s ability to bind to ion channels, such as the Kv1.2 potassium channels, supports the anesthetic effect through modulation of channel gating, which ultimately alters neuronal excitability (Stock et al. 2018; Woll et al. 2017). Enhanced GABAergic transmission contributes to the anesthetic effects by promoting increased inhibition within the CNS, thereby facilitating the induction of a hypnotic state (Choi et al. 2013).

Furthermore, sevoflurane exhibits unique properties that contribute to its analgesic effects. It is proposed that the anesthetic reduces pain sensitivity by modulating gap junction communication between cells and altering action potential thresholds. Additionally, its vasodilatory features may further help in pain management, making it beneficial for procedures requiring analgesia alongside anesthesia (Fernández-Ginés et al. 2022). Other studies have suggested that its central analgesic effects may also result from a direct influence on pain pathways within the brain (DeSousa and Ali 2011), emphasizing sevoflurane’s broad influence in both anesthesia and analgesia.

#### Desflurance

Desflurane, a volatile anesthetic, operates through mechanisms that primarily involve its effects on central nervous system (CNS) neurotransmission and cardiovascular stability. Its anesthetic properties are significantly attributed to its interactions with various neurotransmitter systems, especially gamma-aminobutyric acid (GABA) and N-methyl-D-aspartate (NMDA) receptors. Research has demonstrated that desflurane can induce neuronal cell death via signaling pathways associated with caspase-3-dependent apoptosis and NF-kappaB activation (Liu et al. 2020). This suggests not only an anesthetic effect but also potential neurotoxic effects at high exposure levels.

Moreover, desflurane is recognized for its rapid onset and recovery, qualities resulting from its low blood and fat solubility, which facilitates quick adjustments in anesthesia levels during surgical procedures (Geyik et al. 2021). Its pharmacokinetic profile enables significant gas exchange, making desflurane suitable for low-flow anesthesia, which can reduce overall gas consumption and enhance recovery profiles (Geyik et al. 2021; Barkha et al. 2022). The switch from other agents like sevoflurane to desflurane has also been shown to improve recovery times without worsening hemodynamic responses (Kim et al. 2022; Mikuni et al. 2016).

On the cardiovascular system, desflurane is known for its sympathomimetic effects, which may lead to increased heart rate and blood pressure upon induction due to rapid increments in its concentration during maintenance anesthesia (Kazanci et al. 2009; Lee et al. 2023). This characteristic yields mixed findings regarding its safety in patients with pre-existing cardiovascular conditions; while desflurane can provoke significant cardiovascular stimulation, leading to arrhythmias in some instances, effective management strategies, such as the concurrent use of opioids, can mitigate these risks (Grundmann et al. 2001).

The airway irritation caused by desflurane, especially during induction, has clinical implications. Its pungency can provoke airway reflexes such as coughing and laryngospasm, which are more pronounced during rapid inhalation of the drug (Lee and Jung 2011; Kondo et al. 2020). These reflexive responses underscore the need for careful administration and possibly initial opioid use to suppress hemodynamic perturbations and enhance patient comfort during induction (Kim et al. 2022; Lee and Jung 2011).

Nonetheless, it’s important to acknowledge certain disadvantages associated with the use of inhalant anesthetics in rodent models (Furtado and Andrade 2013). Rapid changes in anesthetic depth necessitate vigilant animal monitoring due to the potential for swift fluctuations, and surgical teams must possess the proficiency to effectively administer and maintain the delivery of inhalant anesthetic agents (Gargiulo et al. 2012). Additionally, the use of a precision vaporizer that is regularly calibrated is indispensable for the accurate administration of inhalant anesthetics, albeit at a higher cost compared with injectable agents. This requirement is particularly relevant for desflurane, which necessitates a dedicated, electronically heated vaporizer with distinct technical specifications, resulting in additional equipment costs and infrastructure demands relative to vaporizers used for other volatile anesthetics (Chakravarti and Basu 2013).

### Reversal agents in laboratory animal anesthesia

The use of specific reversal agents represents an important safety and experimental control strategy in modern laboratory animal anesthesia, particularly when α_2_-adrenergic agonists and benzodiazepines are incorporated into balanced anesthetic protocols. Atipamezole, a highly selective α_2_-adrenergic antagonist, is widely used to reverse the sedative, analgesic, and cardiovascular effects of α_2_-agonists such as xylazine, medetomidine, and dexmedetomidine. Reversal with atipamezole results in rapid restoration of consciousness, improved cardiovascular stability, and shortened recovery times, thereby reducing anesthesia-related morbidity and variability in experimental outcomes (Vainio et al. 1989; Sinclair 2003). Typical dosing of atipamezole is calculated on a molar or milligram basis relative to the administered α_2_-agonist, with species-specific adjustments recommended to avoid abrupt sympathetic rebound.

Flumazenil, a competitive antagonist at the benzodiazepine binding site of the GABA-A receptor, is used to reverse the sedative and muscle-relaxant effects of benzodiazepines such as diazepam and midazolam. Its administration enables rapid recovery while preserving analgesia when benzodiazepines are used as adjuncts to dissociative or injectable anesthetics (Weber et al. 1991). However, flumazenil should be used cautiously in animals with seizure predisposition or in protocols relying on benzodiazepines for anticonvulsant protection.

The cautious use of reversal agents enhances anesthetic safety, facilitates smoother and more predictable recovery, and improves reproducibility in preclinical research. Nevertheless, reversal should be applied selectively and with appropriate monitoring, as abrupt antagonism may unmask pain, induce sympathetic activation, or precipitate adverse cardiovascular responses, particularly following high-dose or prolonged anesthetic exposure (Weber et al. 1991).

## Commonly used anesthetic protocols for laboratory animals

Laboratory animal species vary widely in their response to general anesthetics due to differences in size, metabolic rate, and physiology. Selecting appropriate anesthetic agents and routes is essential for achieving a surgical plane of anesthesia while minimizing adverse effects. Below is an overview of common general anesthesia protocols for mice, rats, rabbits, pigs, and sheep including typical agents, administration routes, dosage ranges, expected anesthesia duration, and species-specific considerations. A comparative summary table is provided at the end for quick reference, see Table 1.

**Table 1:** Comparative summary of general anesthetic protocols for commonly used laboratory animal species, including routes of administration, dosage ranges, primary anesthetic effects, approximate duration of effect, and pertinent practical notes

Species	Anesthetic Agent(s)	Route of Admin.	Dosage Range (units)	Duration of Anesthesia	Primary Anesthetic Effect	Notes
Mouse (rodent)	Ketamine + Xylazine; Isoflurane (inhalant)	IP injection; Inhalation (mask)	Ket: 80–100 mg/kg + Xyl: 5–12 mg/kg; Isoflurane ~ 3–5% (induction), 1–2% (maintenance) (Flecknell P. 2015)	~ 20–30 min (single IP dose); Indefinite with gas (typically hours, with 1–3 min recovery)	K + X: Short-duration surgical anesthesia with limited depth control. Isoflurane: adjustable surgical anesthesia with rapid recovery.	K + X produces short surgical plane (augment with analgesics); Isoflurane allows fine depth control and quick recovery in mice (requires vaporizer, scavenging). High metabolic rate → faster drug clearance, prone to hypothermia.
Rat (rodent)	Ketamine + Xylazine; Isoflurane (inhalant)	IP or IM injection; Inhalation (mask or intubation)	Ket: 40–80 mg/kg + Xyl: 5–10 mg/kg; Isoflurane ~ 2–4% (induction), ~ 1% (maintenance) (Wellington et al. 2013)	~ 60–90 min (single injection); As needed with inhalant (few min induction, rapid recovery)	K + X: moderate to surgical anesthesia of longer duration than in mice. Isoflurane: maintenance anesthesia with fine depth control.	K + X yields ~ 1 h anesthesia in rats (longer than in mice); adding acepromazine can extend duration. Isoflurane is commonly used for maintenance or longer procedures. Rats can be intubated for better airway control; monitor for respiratory depression.
Rabbit	Ketamine + Xylazine (often combined with sedative or opioid); Isoflurane (inhalant)	IM injection (thigh/lumbar); Inhalation via mask or ET tube	Ket: ~20–50 mg/kg + Xyl: 2–10 mg/kg IM (e.g. ~35 mg/kg + 5 mg/kg); Isoflurane ~ 3–5% (induction), 1–3% (maintenance) (Fuentes and Newgren 2008)	~ 20–40 min from IM cocktail (light surgical anesthesia); Inhalant can be maintained for hours (with rapid adjustments)	Injectable protocols: sedation to light surgical anesthesia, often insufficient alone. Inhalants: stable surgical anesthesia for prolonged procedures.	K + X alone often *does not* fully guarantee surgical anesthesia in rabbits, typically used for sedation or induction, then supplemented. Inhalant anesthesia (iso/sevo) is recommended for maintenance of rabbits during major surgery. Use glycopyrrolate (not atropine) if anticholinergic is needed. Intubation is challenging; consider a V-gel or mask. Prevent hypothermia and support respiration (rabbits are sensitive to anesthetic depth).
Pig (swine)	Telazol + Ketamine + Xylazine (TKX cocktail); Isoflurane (inhalant)	IM injection (usually neck); Inhalation via ET tube	Telazol ~ 4–6 mg/kg + Ketamine ~ 2 mg/kg + Xylazine ~ 2 mg/kg IM; Isoflurane ~ 3–4% (induction), 1–2% (maintenance) (Bunnag et al. 2023)	~ 20–30 min surgical anesthesia from TKX IM (sufficient for intubation or minor surgery); Inhalant can be continued as long as needed (minutes to hours)	TKX: rapid immobilization and induction suitable for intubation and short procedures. Inhalants: controlled surgical anesthesia for longer interventions.	TKX (or Telazol + Xylazine) provides rapid sedation and analgesia for intubation and short procedures. For longer surgeries, intubate and use inhalant anesthetic (safer for prolonged depth control). Pigs are prone to malignant hyperthermia with volatile agents (especially certain breeds); avoid halothane, be prepared with dantrolene. Important to fast pigs pre-op (8–12 h) to prevent aspiration. Monitor closely (blood pressure, CO₂, temp); provide ventilation support due to tendency for hypoventilation. Recovery may be slower if long anesthesia or if injectable doses were high, but generally smooth with iso/sevo.
Sheep (ovine)	Ketamine + benzodiazepine; Propofol; Alfaxalone; Tiletamine–zolazepam; Isoflurane/Sevoflurane (inhalant)	IV or IM injection; Inhalation via ET tube	Ketamine ~ 2–5 mg/kg IV (higher IM) ± diazepam/midazolam; Propofol 2–6 mg/kg IV; Alfaxalone ~ 1–2 mg/kg IV; Tiletamine–zolazepam ~ 2–5 mg/kg IV; Isoflurane ~ 1.5–3% (maintenance); Sevoflurane ~ 2–4% (maintenance) (Musk 2024).	~ 10–20 min (injectable induction); Inhalant anesthesia can be maintained for hours	Injectable agents: sedation to short surgical anesthesia suitable for induction. Inhalants: stable, adjustable surgical anesthesia for prolonged procedures.	Balanced anesthesia is recommended. Benzodiazepines improve muscle relaxation and reduce excitatory effects. α₂-agonists (e.g. xylazine 0.02–0.1 mg/kg IV) provide sedation but may cause marked cardiovascular and respiratory depression, particularly in lambs. Adult sheep require prolonged fasting (12–24 h); lambs require minimal fasting to avoid hypoglycemia. Mandatory endotracheal intubation and vigilant monitoring are essential to prevent hypoxemia, hypothermia, and delayed recovery.

### Mice

Mice are small rodents with high metabolic rates, which affect anesthetic dosing and duration. Common general anesthetic approaches in mice include injectable combinations and inhalant anesthetics:

#### ***Injectable Induction***

A combination of ketamine (dissociative anesthetic) and xylazine (α₂-agonist sedative) given intraperitoneally (IP) is widely used. Typical doses are ketamine 80–100 mg/kg and xylazine 5–12 mg/kg IP, yielding about 20–30 min of surgical anesthesia (Kohn et al. 1997) (Table 1). This provides reliable immobilization, though analgesia may be modest; addition of an analgesic (or a tranquilizer like acepromazine) can deepen anesthesia and pain control. Mice anesthetized with ketamine/xylazine should be kept warm (e.g. on a heating pad) due to rapid heat loss and monitored closely because of their fast metabolism and risk of respiratory depression (Kazdağli et al. 2022). Redosing is possible (typically with ketamine alone at a fraction of the initial dose) if more time is needed, but prolonged anesthesia may be better achieved with inhalants to allow finer control (Erickson et al. 2019).

#### ***Inhalant Anesthetics***

Isoflurane delivered via a precision vaporizer (in an induction chamber or nose cone) is commonly used for mice. An induction concentration of ~ 3–5% and maintenance at ~ 1–2% (in oxygen) typically produces surgical anesthesia (Flecknell 2015). Inhalational anesthesia allows easy adjustment of depth and typically has a rapid recovery once the gas is discontinued, owing to isoflurane’s low blood solubility. This method avoids injectable agent metabolism, which is advantageous in mice since their high metabolic rate can lead to quick anesthetic clearance (Flecknell 2015). However, care must be taken to scavenge waste gas and prevent overdose; mice lose consciousness within a few minutes of isoflurane induction (Gargiulo et al. 2012). Because mice are small, intubation is generally not performed; anesthesia is maintained with a nose cone or small chamber (Flecknell 2015). Eye lubricant should be applied to prevent corneal drying, and the animals should be observed until fully recovered (which usually occurs within minutes to tens of minutes after stopping isoflurane) (Greenfield 2019).

#### ***Other Protocols***

Alternatives occasionally used in mice include pentobarbital (a barbiturate) at ~ 50 mg/kg IP, providing ~ 20–40 min of anesthesia. Pentobarbital induces deep anesthesia but has a narrow safety margin and prolonged recovery, so it is generally reserved for non-survival procedures or when inhalants are not feasible (Gaertner et al. 2008). Tribromoethanol (Avertin) at ~ 240 mg/kg IP historically served as a short-acting anesthetic (~ 30 min), but its use is now discouraged due to toxicity and inconsistent effects (Meyer and Fish 2005). Newer agents like alfaxalone (a steroid anesthetic) are being explored; alfaxalone can be given IM or IP to mice, but data on dosing varies (typically a few mg/kg for sedation) (Erickson et al. 2019). In all cases, supplemental analgesia (e.g. buprenorphine or NSAIDs) is recommended for any surgical procedure in mice once they are anesthetized, since general anesthetics like ketamine or isoflurane may not fully suppress nociception (Young et al. 2024).

#### ***Special Considerations***

Mice’s small size means they are prone to hypothermia and dehydration under anesthesia; providing external heat support and fluids (e.g. warm saline IP) during longer procedures is important. Due to their rapid drug metabolism, anesthetic duration is shorter – for instance, a ketamine/xylazine anesthetic plane may only last 20–30 min in a mouse, whereas a similar protocol can last longer in a larger animal. Strain differences exist in anesthetic sensitivity (Gargiulo et al. 2012), so dose adjustments may be needed (what sedates one mouse strain might be insufficient or excessive in another). Mice do not vomit, so risk of aspiration is low, but they should be handled gently to minimize stress (Gargiulo et al. 2012). Recovery is usually fast with inhalants, but one should wait until the righting reflex returns and the mouse is fully alert before returning it to its home cage. Monitoring of respiration (rate, depth) and circulation (e.g. mucous membrane color) is done visually since attaching instruments is challenging on an animal of this size (Gargiulo et al. 2012).

### Rats

Rats, being larger rodents, tolerate anesthetic procedures somewhat differently from mice. They have lower metabolic rates than mice and a greater blood volume, which often results in longer anesthetic durations for a given dose. Common general anesthetic regimens for rats include:

#### ***Injectable Induction***

Ketamine/xylazine is also a staple for rat anesthesia, typically administered IP (or intramuscularly, IM). Doses are usually 40–80 mg/kg ketamine + 5–10 mg/kg xylazine in rats (Wellington et al. 2013). This combination produces about ~ 1 h of surgical anesthesia in rats (approximately 60–80 min of immobilization and unconsciousness) (Gaertner et al. 2008). The duration is longer than in mice because rats metabolize these drugs more slowly. Adding acepromazine (a tranquilizer, e.g. 0.5–1 mg/kg) can prolong the anesthetic effect to ~ 1–2 h total, useful for lengthy procedures (Welberg et al. 2006). As with mice, ketamine/xylazine provides good sedation and moderate analgesia; an opioid or NSAID is often given pre- or intra-operatively for additional pain relief (Sheverini et al. 2024). Rats can be given ketamine/xylazine IM (e.g. into the hindlimb muscles) if IP injection is not ideal, although IM injections may cause irritation. IP route remains common for ease of administration. Deep anesthesia onset is usually within 5–10 min of injection. Monitoring the depth (e.g. loss of pedal reflex, slowed respiration) confirms when the surgical plane is reached (Kazdağli et al. 2022).

#### ***Inhalant Anesthetics***

Rats are frequently anesthetized with inhalants such as isoflurane, similar to mice (Gaertner et al. 2008). An induction of 2–4% isoflurane (in oxygen) and maintenance around ~ 0.5–1.5% is effective. Because rats are larger, they can be anesthetized via a nose cone or face mask; intubation is possible in rats (their trachea can accommodate a small endotracheal tube), and intubation is often done for longer or more invasive surgeries to ensure a protected airway (Zhou et al. 2006). Inhalant anesthesia offers precise control over anesthetic depth and a rapid recovery once discontinued (Rivard et al. 2006). As in mice, an isoflurane-anesthetized rat will recover within minutes to tens of minutes after turning off the vaporizer (depending on duration of anesthesia and gas solubility) (Miller et al. 2016). Respiratory rate and pattern should be observed, and vital signs (heart rate, pulse oximetry if available) should be monitored. Waste gas must be scavenged to protect personnel. In some cases, sevoflurane is used as an alternative volatile agent; it has a slightly faster induction/recovery than isoflurane, though at higher concentrations (e.g. ~4–6% induction for rats) (Smith et al. 1996).

#### ***Other Protocols***

Rats, like mice, can be anesthetized with barbiturates or other injectables (Toon and Rowland 1983). Pentobarbital at 30–50 mg/kg IP was historically common, producing a deep anesthesia for 90–120 min (Ho and Harris 1981), but with very prolonged recovery and significant cardiopulmonary depression (Ren et al. 2012). It is now mainly used for terminal (non-survival) procedures or when inhalants are impractical (Desaulniers et al. 2011). Propofol (an IV anesthetic) can be given to rats via a tail vein to induce anesthesia (doses ~ 5–10 mg/kg IV to effect), usually followed by inhalant maintenance; propofol causes rapid unconsciousness but requires IV access and can cause apnea if given too fast (Trapani et al. 2000 b). Newer agents such as alfaxalone can also be used (either IP or IV in rats, typical induction dose ~ 5–10 mg/kg IV) (Lau et al. 2019). Urethane is an injectable anesthetic used in some physiology experiments for very long-duration anesthesia (several hours from a single IP dose), but it is only for non-recovery use due to carcinogenicity (Field and Lang 1988). As with mice, any surgical procedure in rats under general anesthesia should include analgesics (e.g. buprenorphine 0.01–0.05 mg/kg SC) to manage pain once the animal recovers.

#### ***Special Considerations***

Rats generally handle anesthesia better than mice, they have more robust cardiovascular function and a bit more body mass to tolerate cooling, though they can still become hypothermic and need supplemental heat during anesthesia (Oh and Narver 2024). The larger size of rats permits more options (e.g. intubation, arterial catheterization for monitoring in advanced setups) (Gargiulo et al. 2012). Rats can produce a small amount of saliva during some anesthetic regimes (especially with ketamine/xylazine); an antisialagogue like atropine (0.05 mg/kg SC) or glycopyrrolate can be used to reduce secretions if intubating or if excessive bradycardia is a concern (Cook et al. 1990). However, unlike rabbits, rats do not have high levels of atropine-destroying enzymes, so atropine is effective in this species (Ellinger and Armitage 1953). Fasting is usually not necessary or only brief for rats (they cannot vomit, but an empty stomach helps diaphragmatic movement (Wang et al. 2024). Recovery in rats should be in a warm, quiet area and typically takes 15–30 min for inhalants or 1–2 h for injectable agents to fully wear off (depending on dose) (Oh and Narver 2024). As with mice, strain and age can influence sensitivity, e.g., older rats or certain strains might be more sensitive to xylazine’s depressant effects, so one starts at lower dose ranges and adjusts as needed (Gaertner et al. 2008).

### Rabbits

Rabbits (e.g. New Zealand White rabbits) are frequently used in research and require special considerations for anesthesia (Fuentes and Newgren 2008). They are mid-sized mammals (~ 2–5 kg) with a relatively high metabolic rate (though lower than rodents) and unique physiology (e.g. high sympathetic tone, risk of stress-induced cardiopulmonary issues) (Fuentes and Newgren 2008). Common anesthetic regimens for rabbits include injectable cocktails often followed by inhalant maintenance as well (Wenger 2012).

#### Injectable induction

A ketamine/xylazine combination can be used in rabbits, typically given intramuscularly (in the thigh or lumbar muscles) (Pan et al. 2025). A representative dose is ketamine ~ 35 mg/kg plus xylazine ~ 5 mg/kg IM (dose ranges vary: ~20–50 mg/kg ketamine and 2–10 mg/kg xylazine are reported. This combination produces sedation and immobilization for roughly 20–40 min, sufficient for minor procedures or as induction prior to intubation. However, ketamine/xylazine alone in rabbits may *not* always achieve a deep surgical plane of anesthesia for major surgery (rabbits often retain some reflexes). It provides poor muscle relaxation and if used alone may result in limb or ear movement during painful stimuli. Therefore, ketamine/xylazine in rabbits is commonly used *in conjunction with other agents*: either adding a benzodiazepine (midazolam or diazepam) for better hypnosis (Dupras et al. 2001), or following it with inhalant anesthetic for maintenance. For example, ketamine 15–25 mg/kg + midazolam 2–3 mg/kg IM yields about 30 min of anesthesia and can be reversed (midazolam reversed by flumazenil) for quicker recovery (Gardhouse and Sanchez 2022). Similarly, ketamine + dexmedetomidine (a more specific α₂-agonist) can be used instead of xylazine, often at ~ 15–35 mg/kg ketamine + 0.125–0.25 mg/kg dexmedetomidine IM, producing 30–45 min of anesthesia. Dexmedetomidine is preferred by some veterinarians because xylazine can cause injection site irritation in rabbits (and other species) and tends to produce more bradycardia. After such combinations, the α₂-agonists (xylazine or dexmedetomidine) can be partially reversed with atipamezole or yohimbine to hasten recovery. In summary, injectable anesthetic cocktails in rabbits are useful for short procedures (e.g. imaging, minor surgery) or as a prelude to inhalant anesthesia, but careful monitoring is needed as rabbits are sensitive to anesthetic-induced respiratory depression.

#### Inhalant anesthetics

Inhalation anesthesia is commonly used for rabbits, often after a premedication or induction with injectables to reduce stress. Isoflurane induction typically requires ~ 3–5% in oxygen (delivered by a snug face mask or induction chamber), and maintenance is achieved at **~** 1**–**3%. Sevoflurane, which is less pungent, can also be used (induction ~ 6–8%, maintenance ~ 2–3%) (Terada et al. 2014). Rabbits are prone to breath-holding if placed directly in an anesthetic chamber while awake (due to stress), so pre-sedation (e.g. with acepromazine, midazolam, or an opioid) is advised to smooth the induction. Endotracheal intubation of rabbits is challenging, their narrow mouth, large tongue, and tendency for laryngospasm make it difficult. Skilled personnel may intubate using techniques like a small laryngoscope and lidocaine spray on the larynx (Thompson et al. 2017), or use devices like a supraglottic airway (V-gel) as an easier alternative (Fusco et al. 2021). Once a secure airway is established, inhalant anesthesia can be maintained for extended durations (hours if needed). The rabbit should be ventilated if necessary (spontaneous breathing often continues under moderate isoflurane, but if respirations are shallow or slow, intermittent positive-pressure ventilation can be applied). Recovery from inhalants in rabbits is fairly quick (rabbits usually start waking within minutes of discontinuing isoflurane). They should be kept warm and monitored until they regain the ability to maintain sternal recumbency and swallow. Supplemental oxygen can be given during recovery to ease the transition. Rabbits do not vomit (they lack a vomit reflex), but it is still common to withhold food for a short period (1–2 h) before anesthesia to reduce the risk of regurgitation and to make abdominal organs less distended during procedures. Unlike many species, rabbits must resume eating quickly after anesthesia to avoid gastrointestinal stasis; offering hay or recovery diet as soon as they are alert is recommended (Oglesbee and Jenkins 2012) .

#### Other protocols

Additional injectable anesthetics for rabbits include propofol and alfaxalone (Terada et al. 2014; Tsai et al. 2025). Propofol (a short-acting IV anesthetic) can be given via the marginal ear vein or cephalic vein, a typical bolus is 1–2 mg/kg IV, given slowly to effect (Terada et al. 2014). Propofol induces anesthesia for only a few minutes which is mainly used to facilitate intubation, after which gas anesthesia takes over. It can cause transient apnea, so oxygenation and readiness to intubate are necessary (Tsai et al. 2025). Alfaxalone can also be given IM or IV; for example, 3–5 mg/kg IV or IM produces about 20–30 min of anesthesia in rabbits (Tsai et al. 2025). Alfaxalone often causes less cardiovascular depression and can be useful when inhalant equipment is not available (Sogebi and Cliff 2020). These agents provide flexibility for induction and short procedures but are usually complemented by inhalant anesthesia for longer duration control. In terms of analgesia, rabbits should receive pain relief if a painful procedure is performed. Opioids like buprenorphine (0.01–0.05 mg/kg SC/IV) (Andrews et al. 2020) or nonsteroidal anti-inflammatory drugs (e.g. meloxicam 0.3–0.6 mg/kg SC) are commonly used (Fredholm et al. 2013). It is critical to note that rabbits have a unique metabolic trait: they possess an enzyme called atropinesterase that rapidly metabolizes atropine. Thus, atropine is less effective in rabbits (multiple doses may be needed, or its effects are short-lived) (Harrison et al. 2006). For this reason, glycopyrrolate (0.01–0.1 mg/kg SC/IM) is often preferred as an anticholinergic premedication in rabbits, since glycopyrrolate’s action is longer and not readily broken down by atropinesterase (Olson et al. 1994). If using xylazine or medetomidine in rabbits, one should be cautious of bradycardia and provide an anticholinergic if needed (monitor heart rate; many protocols include glycopyrrolate preemptively) (Raekallio et al. 2002).

Tiletamine–zolazepam is also used in rabbits, primarily for chemical restraint, sedation, and short diagnostic or experimental procedures. Typical doses range from approximately 10–15 mg/kg IM, producing rapid immobilization and moderate anesthesia suitable for brief interventions (Dupras et al. 2001). However, the anesthetic depth achieved with tiletamine–zolazepam can be variable, and recovery may be prolonged or dysphoric, particularly at higher doses (Popilskis and Link 1991). For these reasons, its use in rabbits is generally limited to short, non-painful procedures or as an alternative when other injectable or inhalant anesthetic options are not feasible. Careful monitoring during recovery is recommended, and supplemental analgesia or inhalant anesthesia may be required if deeper or longer-lasting anesthesia is needed (Brammer et al. 1991).

#### Special considerations

Rabbits are obligate nasal breathers, so any obstruction (like mucus or improper head positioning) can cause respiratory distress; ensure the airway is clear and neck extended during anesthesia (Jekl 2021). They also have delicate bones and can injure their spine if struggling, adequate pre-anesthetic sedation helps prevent violent escapes. Stress can cause arrhythmias or hypotension in rabbits, so a calm induction is important (Schadt and Hasser 1998). During anesthesia, rabbits may have a tendency for hypothermia (especially in long procedures), so active warming is needed. Their eyes should be protected (they often have large, prominent eyes that can dry out). Recovery should be in a quiet, warm area; rabbits may take longer to fully recover from injectables (up to an hour or more) if not reversed, whereas recovery from inhalants is quicker. Since rabbits cannot vomit, they do not require extensive fasting (just a few hours of no solid food is typical, primarily to clear the oral cavity of debris), but long fasting should be avoided to prevent hypoglycemia or gut stasis. Post-anesthetic ileus (gastrointestinal slowdown) is a risk in rabbits, so rapid return to feeding and possibly pro-motility drugs might be indicated if appetite does not return. Overall, rabbits require close monitoring of respiratory function (observe chest movements or use a capnograph if intubated) and heart rate (via stethoscope or Doppler) throughout anesthesia, and careful postoperative care.

### Pigs

Pigs (swine) present a very different scale and set of challenges for anesthesia compared to small laboratory animals. Research pigs can range from mini-pigs (~ 20–30 kg) to young farm pigs (50 + kg) or more (Luo et al. 2012). They have a tendency for airway complications and a notable risk of malignant hyperthermia with certain anesthetics (Rosenberg et al. 2015). Common anesthetic strategies for pigs involve a combination of injectable induction and inhalation maintenance.

#### Injectable induction

A well-established approach for anesthetic induction in pigs is the use of Telazol^®^ (a premixed tiletamine-zolazepam combination) combined with other agents such as ketamine and xylazine (Sweitzer et al. 1997). One regimen, often called TKX, involves Telazol 4.4 mg/kg, ketamine 2.2 mg/kg, and xylazine 2.2 mg/kg given IM in the neck or hind limb (Bunnag et al. 2023). This cocktail produces a deep sedation and anesthesia sufficient for intubation and minor surgical procedures. ULAM (University of Michigan) specifically recommends Telazol + xylazine (sometimes with added ketamine) as an excellent IM induction combo, noting it provides *rapid* and reliable sedation for intubation and catheter placement. The onset is about 5–10 min after IM injection. The duration of surgical anesthesia from a Telazol-containing cocktail is on the order of 20–30 min of good effect, after which the pig may begin to lighten (depending on dosing). Adding ketamine to Telazol (to make TKX) increases the dissociative anesthetic component and can reduce the muscle flaccidity or “posterior weakness” seen with Telazol alone (Akaraphutiporn et al. 2024). Another variant is Telazol–Xylazine (TX) without extra ketamine: Telazol dosed in the range of ~ 2–8 mg/kg with xylazine ~ 1–3 mg/kg IM is also used and is noted to cause a bit more ataxia on recovery (hence the addition of ketamine in TKX to improve recovery quality) (Akaraphutiporn et al. 2024). Pigs can also be induced with ketamine + xylazine alone (e.g. ketamine ~ 20 mg/kg + xylazine ~ 2 mg/kg IM), but this may not reliably produce deep anesthesia, it often sedates the pig enough for handling, after which an IV agent or inhalant is used. In any case, once the pig is anesthetized by IM injection, an endotracheal (ET) tube is usually placed to secure the airway. The pig should be intubated cautiously: they have a long soft palate and an angled larynx, making intubation technically difficult; use of a long laryngoscope blade and possibly a stylet or guide tube is helpful. Propofol is another option for induction if IV access is obtained (doses ~ 2–5 mg/kg IV to effect), and thiopental (a barbiturate) historically was used IV (~ 5–15 mg/kg) for rapid induction, both require catheter placement and careful titration to avoid apnea or arrhythmias (Daş et al. 2016; Björkman et al. 1994). After any injectable induction, transitioning to gas anesthesia for maintenance is recommended for longer procedures (Cockshott et al. 1992; Costea et al. 2023).

#### Inhalant anesthetics

For procedures longer than a few minutes, pigs are typically maintained on inhalant anesthetics delivered via the endotracheal tube. Isoflurane is commonly used, at about 3–4% for induction (if masking down a pig, which is often not feasible without pre-sedation) and 1–2% for maintenance (Yasuda et al. 1990). Sevoflurane can be used similarly (often ~ 4–5% induction, 2–3% maintenance), offering a slightly faster induction and recovery than isoflurane (Ishida et al. 2002). Pigs need relatively high fresh gas flows and robust vaporizer settings because of their size and metabolic consumption of oxygen (Jacobsen et al. 2025). While on inhalant anesthesia, pigs should be monitored for ventilatory status, they often require ventilation support due to the depressant effects of the anesthetic and the animal’s large body mass (manual or mechanical ventilation can keep end-tidal CO₂ in check) (Jacobsen et al. 2025). The duration of anesthesia with inhalants can be as long as needed; pigs can safely remain under isoflurane for hours with proper monitoring and support (Haga et al. 2001). Recovery from inhalants is usually rapid once gas is discontinued (pigs will start to swallow and move within minutes to tens of minutes) (Larsen et al. 2021). However, one *critical* risk in pigs is malignant hyperthermia (MH): certain pig breeds possess a genetic mutation that can be triggered by volatile anesthetics (especially halothane, but also isoflurane or sevoflurane in susceptible individuals) and depolarizing muscle relaxants like succinylcholine. MH is characterized by a rapid rise in body temperature, muscle rigidity, acidosis, and cardiovascular collapse. To mitigate this, modern practice avoids halothane in pigs and keeps inhalant concentrations at the minimum required. If a pig is known to be MH-susceptible (e.g. certain lean breeds like Landrace or Pietrain, one might use only injectables or use IV anesthesia (e.g. propofol with opioid infusions – total intravenous anesthesia, TIVA) instead of volatile gas. Additionally, having dantrolene on hand (a muscle relaxant that treats MH) is recommended when anesthetizing pigs that might be at risk.

#### Other protocols

Pigs may also be anesthetized with intravenous infusion techniques. For example, a “triple drip” (GKX) of guaifenesin–ketamine–xylazine can be used as a constant IV infusion for maintenance (Taylor et al. 2008). A typical mixture is 5% guaifenesin with ketamine (2 mg/mL) and xylazine (1 mg/mL) infused at about 2.2 mL/kg/hour after an induction bolus. This IV triple drip provides anesthesia with rapid recovery (~ 30–45 min) once the infusion is stopped. Such techniques are borrowed from large-animal veterinary anesthesia (e.g. in horses) and can be useful for field conditions or when gas anesthesia is not available (Strachan and Welsh 2009). However, for most laboratory settings, inhalant anesthesia via endotracheal tube remains the preferred method for long surgical procedures in swine (Anderson and Mulon 2019). Regarding analgesia, pigs metabolize drugs relatively quickly and often require frequent dosing or CRIs (continuous rate infusions) for pain management (Lervik et al. 2018). Opioids like fentanyl (IV infusion) or buprenorphine (IM injection) and NSAIDs can be used, but attention must be paid to dosing intervals due to shorter half-lives in swine. It’s noted that combining opioids with anesthetics (e.g. fentanyl with isoflurane) can reduce the needed dose of inhalant and stabilize the depth (Strachan and Welsh 2009).

#### Special considerations

Pigs require fasting before anesthesia (food withheld ~ 8–12 h, water allowed up to 2 h prior) because unlike rodents/rabbits, pigs can vomit and are at real risk of aspiration of stomach contents. An endotracheal tube with an inflated cuff helps protect the airway. During anesthesia, pigs are prone to hypoventilation and V/Q mismatch due to their heavy body weight compressing the lungs, hence the need for ventilation support and careful positioning (often in dorsal or lateral recumbency with padding) (Tonge and Robson 2021). Thermoregulation is also an issue: pigs can develop hyperthermia (especially if MH triggers) or hypothermia if the procedure is long; temperature monitoring is essential. Pigs have a robust laryngeal reflex and will laryngospasm if inadequately anesthetized during intubation attempts, using lidocaine spray on the larynx and ensuring a deep plane of anesthesia before intubation is advised (Tsubone et al. 1991). Intra-operatively, monitoring of pigs should include heart rate, blood pressure (if possible), oxygenation (pulse oximetry), and CO₂ (capnography) given their size and the tendency for respiratory depression (Steffey 1986). Arrhythmias can occur; pigs are sensitive to certain catecholamine surges and can develop ventricular arrhythmias if stressed or hypercarbic. Ensuring adequate depth (jaw muscle relaxation is often checked in pigs as a sign of depth and using sedatives pre-emptively (e.g. a benzodiazepine or low-dose ketamine) helps reduce induction stress. On recovery, pigs should be extubated when they show strong swallow reflexes and kept sternal if possible to ease breathing (Loeckinger et al. 2002; Costea et al. 2023). Post-anesthetic myopathies can happen in very large pigs if they’ve been lying on a hard surface for a long time, so padding and occasionally repositioning the animal during anesthesia can help. Overall, pig anesthesia is high-stakes due to the animal’s size and susceptibility to complications, but with balanced protocols (injectable induction + inhalant maintenance + vigilant monitoring), it can be performed safely even for prolonged surgeries.

### Sheep

Sheep are commonly used as laboratory models due to their physiological similarities to humans and their ability to tolerate general anesthesia well. Anesthetic protocols must be tailored to the animal’s age and health, as lambs (young sheep) have important physiological differences from adult sheep. Key considerations include selecting appropriate anesthetic agents, administration routes, dosages, and ensuring a smooth induction, maintenance, and recovery (Costea et al. 2024).

#### Injectable induction

Sheep can be anesthetized using several injectable agents, most commonly as part of balanced protocols. Ketamine is widely used for induction (≈ 2–5 mg/kg IV or higher IM), typically combined with benzodiazepines (diazepam or midazolam) to improve muscle relaxation and reduce excitatory effects, producing short surgical anesthesia (~ 10–20 min) with relatively stable cardiovascular function (Costea et al. 2024). Propofol (2–6 mg/kg IV) is also frequently used for smooth and rapid induction and may be continued as an infusion for maintenance, allowing quick recovery. Alfaxalone (≈ 1–2 mg/kg IV) has emerged as a promising alternative, providing reliable induction with minimal respiratory depression. Tiletamine–zolazepam (≈ 2–5 mg/kg IV) induces rapid anesthesia but may cause prolonged recovery at higher doses. α₂-adrenergic agonists, particularly xylazine (≈ 0.02–0.1 mg/kg IV), are commonly used for sedation in sheep but require caution due to risks of bradycardia, hypotension, and pulmonary edema (Lagutchik et al. 1991). Dexmedetomidine and acepromazine are alternatives for sedation, often combined with opioids (e.g. butorphanol) to enhance analgesia while reducing overall anesthetic requirements (Musk 2024).

#### Inhalant anesthetics

Inhalation anesthesia is the preferred method for maintenance in sheep, especially for prolonged procedures. Isoflurane (MAC ≈ 1.5%) and sevoflurane (MAC ≈ 2.2–2.4%) are the most commonly used agents, typically maintained at 1.5–3% and 2–4%, respectively (Musk 2024). These volatile anesthetics allow precise control of anesthetic depth and rapid recovery due to pulmonary elimination. Induction is usually achieved with injectable agents followed by endotracheal intubation, although mask induction with higher concentrations (e.g. 4–5% isoflurane or 6–8% sevoflurane) may be used when IV access is difficult (Musk 2024). Sevoflurane has been associated with faster recovery compared to isoflurane, particularly in lambs (Vettorato et al. 2012). Nitrous oxide may reduce anesthetic requirements but is generally avoided in ruminants because of ruminal gas expansion and hypoxemia risk. As inhalant agents cause dose-dependent cardiovascular and respiratory depression, continuous monitoring and oxygen supplementation are essential (Musk 2024).

#### Other protocols

Alternative techniques are often employed when inhalation anesthesia is unavailable or impractical. A common example is the “triple drip” (guaifenesin–ketamine–xylazine), administered as an IV infusion to induce and maintain anesthesia with relatively preserved cardiovascular stability (Lin et al. 1993). This protocol is particularly useful in field conditions but still requires airway protection and oxygen supplementation due to hypoxemia risk in ruminants. Total intravenous anesthesia (TIVA) using propofol infusions or intermittent ketamine top-ups may also be used for short to moderate procedures, although drug accumulation can prolong recovery (Dzikiti et al. 2010). In addition, local and regional anesthetic techniques (e.g. lidocaine nerve blocks) are frequently combined with general anesthesia to provide multimodal analgesia. Sheep are sensitive to lidocaine toxicity, so total doses should not exceed ~ 6 mg/kg, with dilution recommended when large volumes are required (Rostami and Vesal 2011).

#### Special considerations

Anesthetic management in sheep must account for ruminant physiology and age-related differences. Adult sheep require prolonged fasting (12–24 h) to reduce rumen volume and aspiration risk, whereas lambs should undergo minimal fasting to avoid hypoglycemia. Lambs have higher metabolic rates, immature hepatic metabolism, limited thermoregulation, and increased susceptibility to hypothermia, necessitating careful dose titration and active warming (Musk 2024). α₂-agonists tend to produce more profound cardiovascular depression in lambs, making benzodiazepine-based protocols preferable in young animals (Kästner 2006). Endotracheal intubation with a cuffed tube is mandatory at all ages to protect the airway, though it may be technically challenging in adult rams due to anatomical constraints (Galatos 2011). Overall, sheep anesthesia relies on balanced protocols, vigilant monitoring, and age-specific adjustments to minimize cardiopulmonary depression, hypothermia, and delayed recovery.

## Depth of anesthesia (DoA) evaluation methods

Accurate assessment of anesthetic depth is essential for maintaining both animal welfare and experimental reliability in preclinical research (Schneider and Sebel 1997). The appropriate depth ensures unconsciousness, immobility, and analgesia during surgical or invasive procedures, while avoiding excessive depression of vital functions. Because anesthetic sensitivity varies widely among species and individual animals, objective and reproducible evaluation methods are required. Over time, a range of approaches have been developed, from traditional reflex- and behavior-based assessments to more sophisticated neurophysiological and autonomic monitoring techniques. The following sections outline these methods in laboratory animals, emphasizing the balance between practicality, invasiveness, and accuracy in determining anesthetic depth across commonly used species such as mice, rats, rabbits, and pigs.

### Traditional reflex-based assessments

Depth of general anesthesia in laboratory animals has historically been evaluated using clinical signs and reflexes (Schneider and Sebel 1997). Observing parameters like respiratory pattern, mucous membrane color, and spontaneous movement provides a basic gauge of anesthetic depth (Joyce et al. 2024). In small rodents (mice and rats), the loss of the righting reflex (inability to correct posture when placed on the back) is a classic indicator of induction of unconsciousness (Teng et al. 2024). During surgical anesthesia, rodents are typically monitored by the absence of purposeful responses to noxious stimuli, such as a firm toe or tail pinch, and by the loss of protective reflexes (Danneman and Mandrell 1997). The most commonly used qualitative tests in rodents include the forelimb and hindlimb pedal withdrawal reflexes, the tail pinch reflex, and the corneal reflex (blink response to corneal touch) (Joyce et al. 2024; Silva et al. 2011). A lack of movement or reflex response indicates a surgical plane of anesthesia, whereas sudden movement or reflexes upon stimulation suggest insufficient depth. Respiratory rate and pattern are also observed: light anesthesia often causes rapid, irregular breathing, whereas a deep plane leads to slow, shallow respirations or apnea. However, changes in breathing alone can be influenced by anesthetic agents and physiology, so they are interpreted in context. In rabbits and pigs, similar reflex-based criteria are applied, for example, checking the jaw tone or ear pinch in rabbits and the response to hoof or limb pinch in pigs, alongside monitoring breathing and muscle tone. In pigs, which frequently undergo immobilization or procedures involving neuromuscular blocking agents, motor responses to stimulation may only serve as indicators of inadequate anesthesia in the absence of complete neuromuscular blockade or during partial recovery from paralysis. When neuromuscular blockade is fully established, assessment of anesthetic depth must rely on alternative indicators, such as autonomic, cardiovascular, and other physiological parameters, rather than motor responses alone (Mirra et al. 2023).

These methods, however, have several limitations including their subjective nature and discontinuous application. Reflexes provide only intermittent “snapshots” of anesthetic depth when tested, and repeated stimulation (e.g. toe pinches) can themselves induce stress or arousal. Moreover, inter-species differences exist: for instance, rodents lose the righting reflex at a relatively light plane of anesthesia (often used to define induction), whereas rabbits can maintain certain reflexes (like corneal reflex) even at surgical depth, necessitating careful interpretation of multiple signs (Henriquez and Evinger 2005). In all these species, reflex absence must be balanced against vital sign trends to avoid overdosing. When available, composite anesthesia scoring systems are sometimes used to integrate multiple reflexes and responses. For example, one study in rabbits employed an anesthesia depth score combining reflex tests and observed behaviors, allowing semi-quantitative tracking of depth over time (Schmid et al. 2025). Overall, reflex-based assessments are indispensable traditional tools with the benefits of immediacy and no equipment, but they are subjective and can be unreliable if used in isolation.

### EEG-based monitoring of brain activity

Advancements in monitoring have enabled the use of electroencephalography (EEG) to assess anesthetic depth in animals, including the development of processed indices like the bispectral index (BIS). In human anesthesia, intraoperative EEG monitors (sometimes called *cerebral function monitors*) are widely used to quantify the hypnotic component of anesthesia. These devices (e.g. BIS, Narcotrend, entropy monitors) distill EEG signals into scalar indices. By definition, BIS values range from 0 (isoelectric EEG, deep anesthesia) to 100 (awake), with a range of 40–60 typically considered adequate anesthesia in humans (Joyce et al. 2024). Translating such EEG monitoring to laboratory animals has both promise and challenges. Small rodents have very high-frequency, low-amplitude EEGs and tiny skull sizes, complicating sensor placement and signal interpretation. Indeed, there are no established clinical EEG monitoring methods for mice or rats during surgery (Joyce et al. 2024). Nevertheless, research teams have demonstrated feasibility: for example, Joyce et al. modified a human BIS monitor with needle electrodes for mice, showing that EEG burst-suppression patterns correlate strongly with higher isoflurane doses (Joyce et al. 2024). In that study, increasing inhalant concentration in mice led to a higher fraction of EEG suppression (isoelectric epochs), indicating deep anesthesia, even when reflexes were absent (Joyce et al. 2024). The processed BIS index itself in mice did not reliably track subtle depth changes, highlighting that algorithms tuned for humans may need adaptation (Joyce et al. 2024). In rats, too, implementing EEG monitors is mainly confined to experimental setups.

For larger species like rabbits and pigs, EEG monitoring is more practical, and numerous studies have evaluated its utility. The BIS monitor is the most investigated EEG-based depth indicator in veterinary research (Petrucci et al. 2023). It has been tested in dogs, cats, rabbits, pigs, horses, goats, and even chickens, with the general finding that anesthetics shift EEG activity toward lower frequencies and higher amplitudes (reflecting unconsciousness) (Petrucci et al. 2023). In rabbits, BIS values tend to decrease upon induction of anesthesia and remain suppressed during maintenance. A recent study of rabbits undergoing neurovascular surgery found that BIS values remained consistently in a low range during a stable surgical plane, and were significantly lower under anesthesia than when waking or at extubation (Petrucci et al. 2023). This suggests that BIS can reflect the anesthetized state in rabbits, at least under balanced anesthesia, with little variation as long as the depth is adequate (Petrucci et al. 2023). Older studies in rabbits under different anesthetics support that processed EEG indices correspond broadly to anesthesia depth: for example, Martín-Cancho et al. reported that BIS values and EEG spectral variables changed predictably with sevoflurane vs. propofol anesthesia in rabbits, in parallel with clinical signs and recovery profiles (Martín-Cancho et al. 2003). However, the experience with BIS in rabbits has not been uniformly positive. Species-specific EEG patterns and technical factors can limit reliability. Notably, a phenomenon of paradoxical BIS increase at very deep anesthesia was reported in rabbits by Romanov et al., where escalating isoflurane beyond the surgical plane caused BIS values to rise unexpectedly despite profound EEG burst suppression (Romanov et al. 2014). The authors attributed this to algorithm artifacts or signal quality issues in extreme EEG depression. Such findings underline that BIS thresholds validated in humans (40–60 for surgery) may not directly apply to animals, in fact, no universal BIS target ranges are established for veterinary species (Petrucci et al. 2023). Nonetheless, when interpreted cautiously, EEG monitors offer a window into the animal’s cortical state that reflexes and autonomic signs alone cannot provide. For pigs, which have gyrencephalic brains more similar to humans, EEG monitoring has been explored extensively. BIS and related measures (e.g. spectral edge frequency 95% [SEF95] and median frequency) have been evaluated during both inhalant- and injectable-based anesthesia in pigs (Mirra et al. 2023). Some studies showed that BIS declines with deeper planes and rises as anesthesia lightens, paralleling clinical endpoints (Mirra et al. 2023). For instance, pigs anesthetized with either sevoflurane or propofol had BIS values that roughly tracked hypnotic depth, but with considerable inter-individual variability (Martín-Cancho et al. 2004). Other studies found *contradictory results*, in a few cases, no significant BIS differences were detected between moderate and deep anesthesia, calling into question its sensitivity (Mirra et al. 2023).

A recent scoping review highlighted that in about half of pig studies investigating BIS, the index did not consistently distinguish depth levels, and overall the evidence for any single EEG-derived indicator in pigs is mixed (Mirra et al. 2023). Artifacts (from muscle activity, surgical cautery, etc.) and differences in EEG dynamics between species contribute to these inconsistencies (Petrucci et al. 2023; Martín-Cancho et al. 2004). Despite these limitations, EEG monitoring in animals provides continuous, objective data on brain function. It is especially valuable when neuromuscular blockers abolish reflexes, a common scenario in large animal surgery where traditional signs vanish. In summary, EEG-based methods (raw EEG observation or processed indices like BIS) are powerful tools for anesthetic depth assessment in lab animals, offering direct insight into the central nervous system depression. Their advantages include quantitative continuous monitoring and potentially early detection of excessively deep anesthesia (e.g. burst-suppression) or impending arousal. The drawbacks are the need for specialized equipment and expertise, and the uncertain translation of index values across species. Calibration or new algorithms may be required to optimize EEG monitors for each animal species. Nonetheless, accumulating evidence suggests that the EEG is highly informative for depth-of-anesthesia monitoring in animals, and research is ongoing to improve its accuracy (Figueroa et al. 2025).

### Autonomic and physiological indicators

Beyond reflexes and EEG, anesthetic depth can be inferred from autonomic responses and other physiological parameters. Anesthesia affects the autonomic nervous system in dose-dependent ways: generally, deeper anesthesia depresses sympathetic tone and blunts stress responses to stimuli, while inadequate anesthesia permits tachycardia, hypertension, or movement in response to pain. Common practice in veterinary anesthesia is to monitor heart rate (HR), blood pressure (BP), respiratory rate, and other signs (such as pupil size or lacrimation in some species) as indirect markers of depth. For example, a sudden increase in HR or arterial BP during surgery often signals that the animal is reacting (suggesting a light plane), whereas profound bradycardia and hypotension may indicate an excessively deep plane (or an anesthetic overdose). These autonomic signs, however, must be interpreted with caution. Physiological variables are influenced by many factors unrelated to anesthetic depth (such as blood volume status, ventilation, drug effects like anticholinergics, etc.) and thus are not specific indicators of consciousness. Indeed, a study in anesthetized animals found that changes in HR and mean BP were not reliable predictors of anesthetic depth when compared to EEG criteria (Thomas et al. 2025). In that study, Thomas et al. (2025) showed that purposeful responses (or their EEG surrogates) could occur without large autonomic swings, and conversely, HR/BP could fluctuate due to surgical reflexes or drug interactions despite stable unconsciousness (Thomas et al. 2025). Veterinary anesthesiologists therefore use trends in vitals alongside other measures, rather than relying on any single parameter.

One advanced approach to glean more depth information from vital signs is heart rate variability (HRV) analysis (Kazdağli et al. 2022). HRV, the beat-to-beat fluctuation in heart rate, reflects the balance between sympathetic and parasympathetic nervous activity. During general anesthesia, the typical shift is toward increased vagal (parasympathetic) influence and reduced sympathetic responsiveness (Kazdağli et al. 2022). In principle, a reduction in HRV can indicate a deep anesthetic state with suppressed autonomic responsiveness, whereas higher HRV might suggest lighter anesthesia or adequate analgesia allowing normal autonomic oscillations. Researchers have proposed HRV-based indices as complementary depth monitors, on the premise that autonomic tone correlates with the level of hypnosis and antinociception (Wujtewicz and Owczuk 2023). For instance, the Analgesia Nociception Index (ANI), derived from high-frequency HRV components, has been explored in some animal studies as a real-time gauge of nociceptive balance under anesthesia (Picker et al. 2001). In mice and rats, HRV analysis has been used experimentally to compare anesthetic regimes: one study showed different anesthetic agents produce distinct HRV profiles in mice, corresponding to their depressive effects on sympathetic outflow (Kazdağli et al. 2022). However, the application of HRV in small animals is constrained by their high basal heart rates and the need for high-fidelity ECG recordings. In larger species, HRV monitoring is more feasible; for example, HRV has been studied in anesthetized cats and pigs as a research tool to indicate depth or analgesic adequacy (Picker et al. 2001). Autonomic indicators such as HRV are attractive because they are noninvasive and continuous, but they are highly sensitive to confounders (e.g. drugs like atropine obliterate meaningful HRV signals, and surgical stimuli or ventilation can alter HRV independently of depth) (Pattanapon et al. 2018). Therefore, HRV is still considered an adjunct monitoring modality.

Other physiologic monitors can also inform depth assessment. Respiratory rate and end-tidal CO₂ trends may show central depression at deep planes (e.g. slow shallow breathing, rising CO₂), versus hyperventilation or breath-holding if an animal is too light. In species like rabbits that are prone to stress, observing the pattern of breathing (smooth vs. irregular gasps) can alert to arousal (Ryu et al. 2016). Temperature can indirectly relate to depth as well, since very deep anesthesia can lead to hypothermia due to reduced metabolic rate (though maintaining normothermia is a standard supportive care, not a depth monitor per se) (Redfors et al. 2014). In pigs and other large animals, cerebral oximetry or brain perfusion monitors (such as near-infrared spectroscopy) have occasionally been used in research to detect changes in brain state that might correlate with depth, especially during low-flow states or hemorrhagic models (Mirra et al. 2023; Silva and Antunes 2012). While not routine for depth monitoring, these modalities underscore the broad range of physiologic changes that accompany different anesthetic planes.

**Species-specific considerations** are important when relying on autonomic signs. Rodents have such rapid basal heart and respiratory rates that significant changes are needed to be noticeable, and they often receive no invasive monitoring; specialized miniature sensors are available (e.g. tail-cuff blood pressure, pulse oximetry for rodents), but these are less sensitive and rarely used intraoperatively (Joyce et al. 2024). Rabbits commonly exhibit stress-induced arrhythmias or bradycardia under anesthesia; many protocols include anticholinergic premedication to stabilize heart rate, which in turn means HR is not a useful indicator of depth (since it’s pharmacologically fixed) in those cases. Instead, rabbit anesthetists might monitor parameters like ear pinch reflex or jaw tone in addition to breathing and EEG/BIS if available (Petrucci et al. 2023; Romanov et al. 2014). Pigs have cardiovascular dynamics closer to humans and typically are instrumented with arterial lines and ECGs during research anesthesia – providing continuous BP and HR data. As noted, though, pigs under surgical anesthesia may not show marked autonomic responses unless the stimulus is substantial, especially if opioid analgesics are used. A scoping review of pig anesthesia methods emphasized the lack of any single reliable autonomic or EEG measure and recommended using a combination of clinical signs and, when possible, brain monitors to avoid unintentional overdose or awareness (Mirra et al. 2023).

In conclusion, assessing the depth of general anesthesia in mice, rats, rabbits, and pigs requires a multimodal approach that balances traditional reflex tests with advanced monitoring techniques. Reflex and clinical sign assessment is fundamental and species-adapted – from the righting reflex and pedal withdrawal in rodents to palpebral or corneal reflex and movement in rabbits and pigs – offering simplicity but limited precision. EEG-based monitoring, including raw EEG interpretation and indices like BIS, represents a significant advance, providing direct insight into brain anesthetic effects; its use has shown promise (especially in larger animals) but demands careful species-specific validation and awareness of its limitations (such as algorithm artifacts or lack of defined index targets in animals). Autonomic and ancillary measures (heart rate, blood pressure, HRV, breathing patterns, etc.) continue to serve as useful supplemental indicators of depth, with the understanding that they reflect the balance between anesthetic suppression and physiological reactivity. Each approach comes with advantages, from ease of use to objective quantification – and drawbacks, from subjectivity to technical complexity. Therefore, veterinarians and researchers typically integrate multiple monitoring modalities to ensure laboratory animals are maintained at an appropriate anesthetic depth: deep enough to prevent pain and awareness, yet not so deep as to jeopardize cardiovascular stability or recovery. By appreciating the species-specific responses and using tools ranging from a simple toe pinch to sophisticated EEG monitors, one can achieve a more reliable evaluation of anesthetic depth in these animal models, ultimately improving both animal welfare and experimental rigor.

## Conclusions

General anesthesia in laboratory animals is not a neutral backdrop but an active biological influence that shapes autonomic, cardiovascular, neurological, and biochemical readouts. Across species, commonly used injectable (e.g., ketamine–α_2_ agonists, barbiturates, propofol) and inhalational agents (isoflurane, sevoflurane, desflurane) differ in mechanisms, depth profiles, and side-effect burdens that can bias experimental outcomes if not anticipated. Practical, species-tailored protocols exist for mice, rats, rabbits, and pigs, yet their safe and reproducible use requires vigilant monitoring. No single depth-of-anesthesia metric is sufficient: traditional reflex testing should be integrated with continuous physiological monitoring and, when feasible, EEG-based measures, acknowledging species-specific limitations of processed indices. Investigators should predefine anesthetic plans aligned with experimental endpoints, provide multimodal analgesia, maintain normothermia and ventilation, and report anesthetic details transparently. Future work should refine species-validated EEG algorithms, standardize autonomic indices (including HRV) for intraoperative decision-making, and expand awake paradigms where appropriate. Thoughtful anesthetic selection and multimodal depth assessment will enhance animal welfare and strengthen the validity and reproducibility of preclinical research.

## Data Availability

No datasets were generated or analysed during the current study.
